# Identification, antimicrobial susceptibility test, and pathogenicity analysis of *Shigella flexneri* from cynomolgus monkey (*Macaca fascicularis*)

**DOI:** 10.3389/fmicb.2026.1907731

**Published:** 2026-08-20

**Authors:** Ziyao Qian, Heling Li, Long Zhang, Zhigang Chen, Faliang Zong, Hong Wang

**Affiliations:** 1 State Key Laboratory of Primate Biomedical Research, Institute of Primate Translational Medicine, Kunming University of Science and Technology, Kunming, Yunnan, China; 2 Yunnan Key Laboratory of Primate Biomedical Research, Kunming, Yunnan, China; 3 Kunming Biomed International Ltd., Kunming, Yunnan, China

**Keywords:** antimicrobial susceptibility test, cynomolgus monkey, identification, pathogenicity analysis, *S. flexneri*

## Abstract

**Introduction:**

*Shigella flexneri* (*S. flexneri*) is an important zoonotic pathogen. Infections caused by this bacterium in nonhuman primates, especially cynomolgus monkeys (*Macaca fascicularis*), represent major health risks and biosecurity concerns for laboratory animal facilities. Characterizing *S. flexneri* isolates originating from captive cynomolgus monkeys is essential for implementing targeted disease prevention and control strategies.

**Methods:**

In this present study, strain MF041021 was isolated from fecal samples of a dysenteric cynomolgus monkey. Species identification was accomplished using 16S rRNA gene sequencing. Additional phenotypic and genotypic characterizations included detection of virulence genes, antimicrobial susceptibility testing against clinically important agents, and screening for antimicrobial resistance genes. Pathogenicity of the isolate was evaluated via intraperitoneal inoculation in C57BL/6 mice.

**Results:**

The isolate MF041021 (GenBank accession: PZ779204) was identified as *S. flexneri* by 16S rRNA sequencing. Sequence analysis revealed that the 16S rRNA gene of this isolate shared over 97.1% similarity with those of ten *S. flexneri* strains derived from various hosts deposited in GenBank, with the highest similarity (99.9%) observed with a human-derived strain (CP050985). Virulence gene profiling demonstrated that the isolate harbored four virulence-associated genes (*ipaC, ipaH, ial*, and *shet1A*), while the sen gene was absent. Pathogenicity was pronounced in C57BL/6 mice. Antimicrobial susceptibility testing showed that the isolate was resistant to ampicillin but susceptible to ciprofloxacin, imipenem, meropenem, levofloxacin, cotrimoxazole, and ofloxacin. Resistance gene analysis identified *tetB, ampC, ant (3*′*)-Ia*, and *aadA1*.

**Discussion:**

The cynomolgus monkey-derived *S. flexneri* isolate MF041021 demonstrated substantial pathogenic potential, harboring multiple virulence factors and antimicrobial resistance genes. Its close phylogenetic relationship with human-derived strains, as evidenced by 16S rRNA gene analysis, underscores the need for enhanced biosecurity, standardized husbandry practices, and prudent antimicrobial stewardship in laboratory macaque colonies. These findings from this single clinical isolate provide valuable case-level reference data for the diagnosis, prevention, and control of *S. flexneri* infection in captive non-human primates.

## 1 Introduction

*Shigella flexneri*, a member of the genus *Shigella*, is a Gram-negative, short rod-shaped bacterium. It is characterized by its non-encapsulated, non-spore-forming, non-flagellated, and non-motile; however, it possesses fimbriae and functions as a facultative anaerobe. As one of the primary causative agents of bacillary dysentery (commonly referred to as shigellosis), this pathogen is widespread globally, particularly in developing countries with low socioeconomic status. It accounts for approximately 125 million episodes of diarrhea and around 160,000 deaths annually, including an estimated 53,000 children (Bardhan et al., 2010). Shigellosis typically presents as a self-limiting condition (Schroeder and Hilbi, 2008), with both patients and carriers serving as the sources of infection primarily transmitted via the fecal-oral route. Experimental infections have demonstrated that as few as 10 bacterial cells can induce disease in adults (Toro et al., 2015), indicating an exceptionally high level of infectivity. Infected individuals may exhibit symptoms ranging from watery diarrhea to severe dysentery, including fever, abdominal pain, diarrhea, mucopurulent bloody stools, and prostration; toxic shock may occur in severe cases (Shooraj et al., 2024). Although the mortality rate associated with shigellosis has significantly decreased over the past three decades, its incidence remains elevated alongside extraintestinal complications resulting from recurrent moderate to severe diarrhea and dysentery. These complications may encompass malnutrition, growth retardation, and impaired cognitive development (Hosangadi et al., 2019).

The cynomolgus monkey (*Macaca fascicularis*), a member of the genus Macaca, is primarily distributed across Thailand, Laos, Vietnam, Malaysia and other countries (Balansard et al., 2019). Due to its physiological similarity to humans, strong adaptability, and ease of husbandry, it has become an established and widely utilized non-human primate model in biomedical research. Shigellosis represents a major enteric disease of captive nonhuman primates (NHPs) and is also particularly prevalent in macaques used for biomedical research. A multicenter case-control study demonstrated a significant association between fecal *Shigella* isolation and diarrhea in rhesus macaques, with a high attributable risk, corroborating *Shigella* as a key diarrheal pathogen in this species (Haertel et al., 2025). Consistent with this, Maxwell et al. likewise identified *Shigella* as a common enteric pathogen in captive NHP populations (Maxwell et al., 2022). Transmission occurs predominantly via the fecal-oral route and may be facilitated by group housing, direct or indirect animal-to-animal contact, and environmental fecal contamination. In a well-managed research facility, Mannion et al. screened 24 macaques and recovered S. flexneri from three individuals, corresponding to a culture-positivity rate of 12.5%, indicating that localized transmission may occur even under standard husbandry conditions (Mannion et al., 2018). More recently, an outbreak of multidrug-resistant *S. flexneri* serotype 2a at a zoological facility in the United States affected 15 of 25 NHPs, yielding a clinical attack rate of 60%; four affected animals succumbed to infection, corresponding to an apparent case-fatality ratio of 26.7% (Shrum Davis et al., 2025). Clinically, shigellosis may present as acute dysentery or recurrent diarrhea and can be fatal in severe cases. Beyond direct morbidity and mortality, the disease necessitates increased diagnostic screening, animal isolation, environmental decontamination, and antimicrobial therapy guided by susceptibility testing. In research colonies, *Shigella* infection and its treatment may alter baseline health and physiological parameters, thereby introducing potential confounding effects that compromise the interpretation of experimental outcomes. Accordingly, strengthened pathogen surveillance, environmental hygiene, biosecurity measures, and routine antimicrobial susceptibility testing are essential for safeguarding both animal welfare and research integrity. The complex and variable serotypes of *Shigella*, coupled with the increasingly severe problem of antimicrobial resistance, have brought great challenges to clinical treatment (Anderson et al., 2016; Puzari et al., 2018). To date, research on *Shigella* infection in experimental monkeys, including *S. flexneri*, has established a relatively systematic scientific framework. This framework not only serves as a cornerstone for elucidating fundamental pathogenic mechanisms but also provides a pivotal translational medicine platform for the development of vaccines and drugs (Barnoy et al., 2011). Although previous studies have described the etiology, pathogenesis, and experimental model applications of *Shigella* infection in NHPs, there remains a paucity of comprehensive case-based data integrating the phenotypic characteristics, virulence gene profiles, antimicrobial resistance determinants, genotype–phenotype relationships, and pathogenic potential of *S. flexneri* isolates recovered from clinically diseased captive cynomolgus monkeys. In laboratory macaque colonies, the systematic characterization of a single clinical isolate cannot establish prevalence or transmission dynamics; nevertheless, it provides valuable reference data to inform clinical diagnosis, biosecurity isolation protocols, antimicrobial treatment decisions, and future molecular epidemiological surveillance. Therefore, the present study performed an integrated characterization of an *S. flexneri* isolate recovered from a dysenteric cynomolgus monkey, employing morphological observation, biochemical identification, serological testing, 16S rRNA sequencing, targeted virulence and antimicrobial resistance genes detection, antimicrobial susceptibility testing, and a mouse pathogenicity assay.

The present study aims to generate comprehensive case-level characterization data for this isolate, providing preliminary evidence and practical reference to inform the etiological identification, clinical management, infection control, and subsequent high-resolution genomic surveillance of *S. flexneri* infections in laboratory-housed monkeys.

## 2 Materials and methods

### 2.1 Samples

The cynomolgus monkey used in this study was sourced from Kunming University of Science and Technology [SCXK (Yunnan) 2022-0001]. The diseased animal was a 22-year-old male cynomolgus monkey (*Macaca fascicularis*) housed individually. No antimicrobial agents had been administered during the week preceding disease onset, and the animal had no known history of chronic underlying disease. Primary clinical symptoms in diseased monkey observed included acute fever, lethargy, and complete anorexia, accompanied by dysentery characterized by mucopurulent bloody stools and tenesmus. Additional signs included nausea, vomiting, decreased skin elasticity, sunken eyeballs, and persistent lethargy. Following disinfection of the anus and perianal skin with iodophor, a sterile disposable sampling swab moistened with sterile normal saline was utilized to collect rectal fecal samples. Subsequently, the samples were promptly transported to the laboratory for parasitological examination and bacterial culture. To assess the health status of cohoused neighboring animals, rectal swabs were additionally collected from five monkeys housed in adjacent cages 24 h following index case sampling. All animals within the same housing room were subsequently monitored clinically for 3 consecutive days, with daily recording of diarrhea incidence and any other abnormal clinical manifestations.

### 2.2 Bacterial isolation and morphological observation

Freshly collected fecal swabs were placed in 1 mL of sterile saline and thoroughly vortexed to obtain a homogeneous suspension. A loopful of the suspension was inoculated onto MacConkey (MAC) agar and Salmonella-Shigella (SS) agar plates using the quadrant streak plate method, followed by incubation at a constant temperature of 37 °C for 18–24 h. Presumptive *Shigella* colonies were selected based on their typical morphology on MAC and SS agar plates with colorless, translucent, smooth circular colonies, moist surfaces and absent black centers. Suspected single colonies were picked in a biosafety cabinet, inoculated onto SS agar medium and triple sugar iron (TSI) slant medium, and incubated at 37 °C for an additional 18–24 h for further observation and confirmation. The suspected single colonies were smeared onto glass slides, fixed, subjected to Gram staining, and observed under a light microscope using a 100 × oil immersion objective to assess bacterial morphology and staining characteristics.

### 2.3 Physiological and biochemical identification

The isolated and purified strain was diluted with normal saline to prepare a bacterial suspension with turbidity equivalent to the 0.5 McFarland standard. 50 μL of this bacterial suspension was added to micro biochemical identification tubes and incubated at a constant temperature of 37 °C for periods ranging from 18 to 72 h. Following incubation, color changes in the tubes were observed; the biochemical characteristics of the isolated strain were determined according to manufacturer instructions. Most biochemical reactions were read following 18 24 h of incubation, whereas tests requiring extended incubation were recorded at 48 or 72 h.

### 2.4 Serological diagnosis and typing of the isolated strain

A clean glass slide was divided into two regions, labeled as “test region” and “control region” respectively. A drop of polyvalent diagnostic serum specific for the *Shigella* genus was placed in the test region, while a drop of normal saline was added to the control region. Subsequently, a suspected single colony was selected from the culture medium using an inoculating loop and mixed with the diagnostic serum. The results were evaluated against a black background: within 1 min, pronounced granular agglutination appeared in the test region, indicating a positive reaction; conversely, no agglutination occurred in the control region, which confirmed a negative reaction characterized by uniform turbidity without any signs of agglutination.

### 2.5 16S rRNA sequencing and phylogenetic analysis of the isolated strain

Although 16S rRNA gene sequencing has certain limitations in discriminating between closely related bacteria species and providing high-resolution phylogenetic analysis, it remains a standard and most widely used first-line method for routine bacterial species identification. In this study, 16S rRNA gene sequencing was applied in combination with morphological, biochemical, and serological approaches to achieve robust, comprehensive species identification. Primer sequences: 27F:5′-AGAGTTTGATCCTGGCTCAG3′; 1492R:5′-GGTTACCTTGTTACGACTT3′. These primers were synthesized by Beijing Tsingke Biotechnology Co., Ltd. The PCR reaction system (25 μL) consisted of 1 μL each of forward and reverse primers (10 μmol/L), 12.5 μL of 2 × Taq PCR Mix, and 10.5 μL of ddH_2_O. A small amount of a suspected single colony was picked with a sterile pipette tip, thoroughly mixed in the above system through pipetting, and used as the PCR template for amplification. The PCR reaction procedure included an initial denaturation at 94 °C for 3 min; followed by 35 cycles comprising denaturation at 94 °C for 30 s, annealing at 55 °C for 30 s, and extension at 72 °C for 1 min; final extension at 72 °C for 5 min; storage was performed at 4 °C. 5 μL of the PCR product underwent electrophoresis on a 1.5% agarose gel to evaluate DNA integrity, purity, and fragment size. Qualified amplification products were purified and recovered before being sent to Beijing Tsingke Biotechnology Co., Ltd. for sequencing.

The obtained gene sequence was subjected to BLAST homology alignment with related sequences published in GenBank utilizing the MegAlign software from the DNAStar software package. A phylogenetic tree was constructed using MEGA11 software to analyze and compare the genetic relationships of *S. flexneri* strains from various sources.

### 2.6 Detection of virulence and resistance genes of the isolated strain

Total DNA of the isolated strain was extracted using a bacterial genomic DNA extraction kit. To further analyze the pathogenicity-related genes as well as resistance-associated genes of the isolated strain, five virulence genes (*ipaC, ipaH, ial, sen, shet1A*) and a total of 14 resistance genes in four categories (including tetracyclines, β-lactams, sulfonamides and aminoglycosides) were selected for PCR detection. The primer sequences were obtained from the literature (Karimi-Yazdi et al., 2020; Sangeetha et al., 2014; Talukder et al., 2007; Peng et al., 2021; Ng et al., 2001; Mazel et al., 2000; Maidhof et al., 2002) and synthesized by Beijing Tsingke Biotechnology Co., Ltd. (Table 1). The PCR reaction system and amplification procedures were the same as described in 2.5. Subsequently, amplified products were analyzed via 1.5% agarose gel electrophoresis.

**Table 1:** Primers for virulence and resistance genes.

Classification	Genes	Primer sequences 5^′^-3^′^)	Fragment length/bp	Annealing temperature/°C
**Virulence genes**	*ipa C*	F:GCGAGGATCCATGGAAATTCAAAACACA	1,112	60
		R:GAGAGTCGACGTTAAGCTCGAATGTTAC		
	*ipa H*	F:TGGAAAAACTCAGTGCCTCT	423	55
		R:CCAGTCCGTAAATTCATTCT		
	*shet1A*	F:TCACGCTACCATCAAAGA	309	55
		R:TATCCCCCTTTGGTGGTA		
	*sen*	F:ATGTGCCTGCTATTATTTAT	799	55
		R:CATAATAATAAGCGGTCAGC		
	*ial*	F:CTGGATGGTATGGTGAGG	320	55
		R:GGAGGCCAACAATTATTTCC		
**Drug resistance genes**	*tetA*	F:GCTACATCCTGCTTGTGCCTTC	210	57
		R:CATAGATCGCCGTGAAGAGG		
	*tetB*	F:TTGGTTAGGGGCAAGTTTTG	659	54
		R:GTAATGGGCCAATAACACCG		
	*ampC*	F:TTCTATCAAMACTGGCARCC	530	55
		R:CCYTTTTATGTACCCAYGA		
	*bla_*TEM*_*	F:GTCGCCGCATACACTATT	365	53
		R:CGCCTCCATCCAGTCTAT		
	*bla_*NDM*_*	F:TGACGCGGCGTAGTGCTCAGTGT	297	60
		R:GCGGCGGGGATTGCGACTTAT		
	*sul1*	F:GGTTCTGAAATCCATCCCTG	251	56
		R:CTCTCATCGAAGAAGGAGTC		
	*sul2*	F:CGGCATCGTCAACATAACC	369	57
		R:CCGAATGCATAACGACGAG		
	*sul3*	F:AAACGAATCCGGAAGAGGT	279	57
		R:CAAGAGTTGGTGCTAAACGA		
	*ant(3′)-Ia*	F:ATCTGGCTATCTTGCTGACA	284	49
		R:TATGACGGGCTGATACTGG		
	*aph(3′)-IIa*	F:TGACTGGGCACAACAGACT	717	50
		R:TCAAGAAGGCGATAGAAGGC		
	*aac(3′)-II*	F:TGAAACGCTGACGGAGCCTC	370	54
		R:GTCGAACAGGTAGCACTGAG		
	*rmtB*	F:ATGAACATCAACGATGCCCTC	756	49
		R:TTATCCATTCTTTTTTATCAAGTATAT		
	*strA-strB*	F:CCAATCGCAGATAGAAGGCAAG	580	65
		R:ATCAACTGGCAGGAGGAACAGG		
	*aadA1*	F:GCAGCGCAATGACATTCTTG	282	54
		R:ATCCTCGGCGCGATTTTG		

### 2.7 Antimicrobial susceptibility testing

Antimicrobial susceptibility testing was performed on the fecal *Shigella* isolate against five clinically relevant antimicrobials including ampicillin, ciprofloxacin, levofloxacin, ofloxacin, and cotrimoxazole. Given the clinical importance of carbapenems for treating multidrug-resistant enterobacterales infections, imipenem and meropenem were additionally included as exploratory agents. Gentamicin was also tested to assess aminoglycoside susceptibility and to enable genotype–phenotype correlation analyses in conjunction with resistance gene profiling. The results obtained for imipenem, meropenem, and gentamicin were used solely for exploratory analyses and were not intended to inform routine clinical treatment recommendations.

Antimicrobial susceptibility testing was performed using the Kirby–Bauer disk diffusion method. The concentration of the bacterial suspension in the logarithmic growth phase was adjusted to 0.5 McFarland turbidity using the McFarland turbidimetry method. 100 μL of the bacterial suspension was evenly spread on Mueller-Hinton (MH) agar medium under sterile conditions. The antimicrobial susceptibility disks intended for testing were placed on the surface of the MH medium with sterile forceps, and the plates were incubated at 35 ± 2 °C in ambient air for 16–18 h. Following incubation, the diameter of each inhibition zone was measured in millimeters using a digital caliper. Isolates were categorized as susceptible (S), intermediate (I), or resistant (R) according to the interpretive criteria specified in the Clinical and Laboratory Standards Institute [Clinical and Laboratory Standards Institute (CLSI), 2025 guidelines].

### 2.8 Animal pathogenicity testing

Animal preparation: 15 SPF-grade C57BL/6 mice purchased from Shanghai Model Organisms Center, Inc., were randomly divided into three groups (two experimental groups and one control group), with five mice allocated to each group.

Bacterial preparation: the isolated and purified *S. flexneri* strain (MF041021) was inoculated into 50 mL of Luria-Bertani (LB) liquid medium and incubated at 37 °C for 12 h to facilitate enrichment. Bacterial cells were collected by centrifugation at 4,000 rpm for 10 min, after which supernatant was discarded. The bacterial concentration of the stock suspension was determined by plate counting to be 4.67 × 10^8^ CFU/mL. An appropriate aliquot of the stock suspension was then diluted 100-fold with sterile phosphate-buffered saline (PBS) to obtain a suspension at 4.67 × 10^6^ CFU/mL. These two bacterial suspensions were used for the high-dose and low-dose groups, respectively. An intraperitoneal injection mouse model was adopted to preliminary assess the systemic virulence and *in vivo* infectivity of the *S. flexneri* isolate. This model features straightforward operation and high reproducibility, rendering it ideal for preliminary screening of bacterial invasive capacity. Each mouse in both experimental groups received an intraperitoneal injection of either 0.5 mL of PBS bacterial suspension at different concentrations; meanwhile, each mouse in the blank control group received an equal volume of sterile PBS via intraperitoneal injection as well. Mice were maintained under normal feeding conditions following challenge; their clinical signs including locomotor activity and general condition were continuously monitored within 12 h. In instances where any mice succumbed during this observation period, immediate dissection ensued to assess pathological changes; additionally, re-isolation and identification procedures for bacteria from the liver and cardiac blood samples were conducted.

## 3 Results

### 3.1 Morphological characteristics of the bacterium

**Figure 1:**
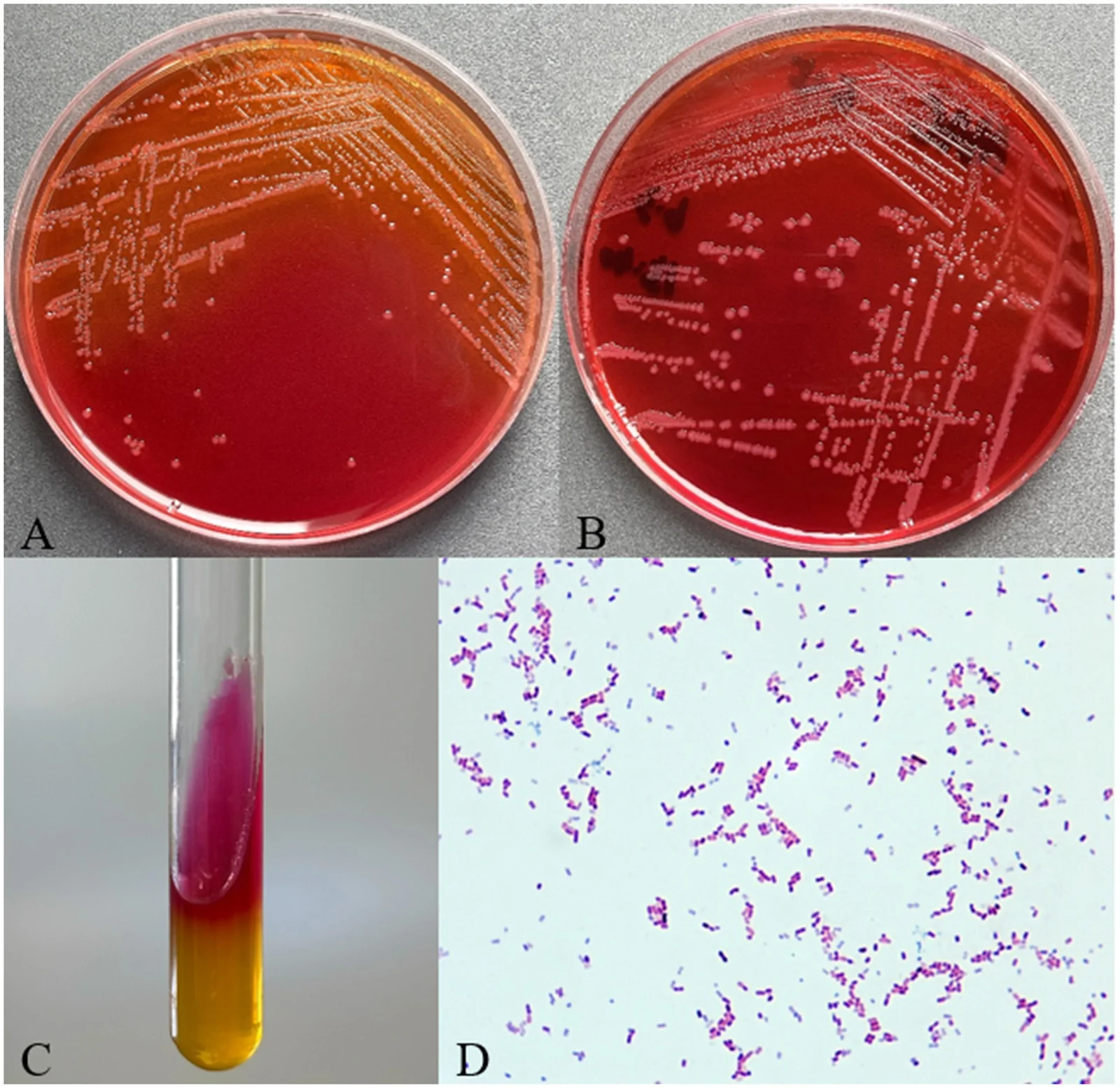
Morphological characteristics of isolated bacteria. **(A)** Morphological observations of isolated bacteria on SS medium. **(B)** Morphological observations of isolated bacteria on MAC medium. **(C)** Morphological observations of isolated bacteria on TSI agar medium. **(D)** Gram staining of the isolated strain (100 × ).

A suspected strain of *Shigella* was isolated from the fresh feces of a cynomolgus monkey exhibiting pronounced diarrhea symptoms. Following incubation on SS medium (Figure 1A) and MAC medium (Figure 1B) for 24 h, only one type of colony emerged on both media. The colonies were slightly elevated, smooth, moist, with sharp edges, colorless and translucent, circular in shape, measuring approximately 1 and 2 mm in diameter. Fading and yellowing occurred around the colonies growing on MAC and SS media. Subsequent culturing on TSI agar medium for additional 24 h, the slant was alkaline and red, the butt was acidic and yellow without gas or hydrogen sulfide production (Figure 1C). Gram-stained of the colonies indicated uniform Gram-negative short rods (Figure 1D). The isolated strain was designated as MF041021. In contrast, no bacterial growth was detected after a similar culture period using fecal samples from animals housed in adjacent cages. None of the five monkeys housed in adjacent cages exhibited diarrhea or other apparent clinical abnormalities throughout the 3-day observation period, and *S. flexneri* was not isolated from any of their rectal swab specimens.

### 3.2 Results of bacterial biochemical identification

Biochemical testing demonstrated that the isolated strain produced acid without gas formation, did not generate hydrogen sulfide, and exhibited no motility. Mannitol and MR tests yielded positive results while all other indicators were negative (Table 2). According to criteria outlined in the Manual of Systematic Identification of Common Bacteria, these biochemical characteristics align with those typical of *S. flexneri*.

**Table 2:** Biochemical reaction results of isolated bacteria.

Biochemical project	Results	Biochemical project	Results
Mannitol	**+**	Semi-solid agar (motility)	**–**
Hydrogen sulfide (H_2_S)	**–**	Glucose (gas)	**–**
Phenylalanine	**–**	Lysine	**–**
Gluconate	**–**	Ornithine	**–**
Indole	**–**	Raffinose	**–**
Voges–Proskauer test	**–**	Sorbitol	**–**
Methyl red test (MR test)	**+**	Scutellarin	**–**
Citrate	**–**	Xylose	**–**
Urea	**–**		

“+”indicated positive; “–”indicated negative.

### 3.3 Serological diagnosis of the isolated strain

**Figure 2:**
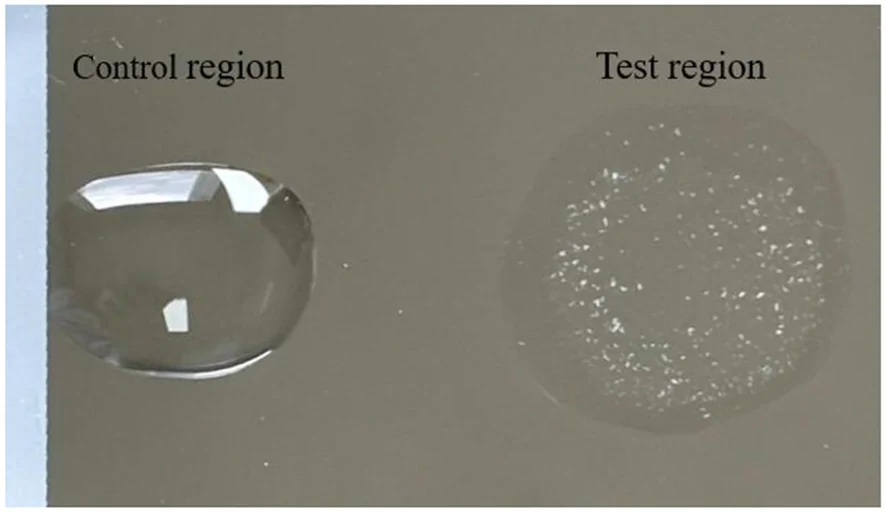
Serological diagnostic results for the isolated strain.

Upon mixing the suspected strain with polyvalent diagnostic serum to *Shigella* genus, significant granular agglutination occurred within 1 min in the test region; however, no agglutination was observed in the control region (Figure 2), indicating a positive reaction of the isolated strain.

### 3.4 16S rRNA gene sequencing, homology comparison, and phylogenetic analysis

**Figure 3:**
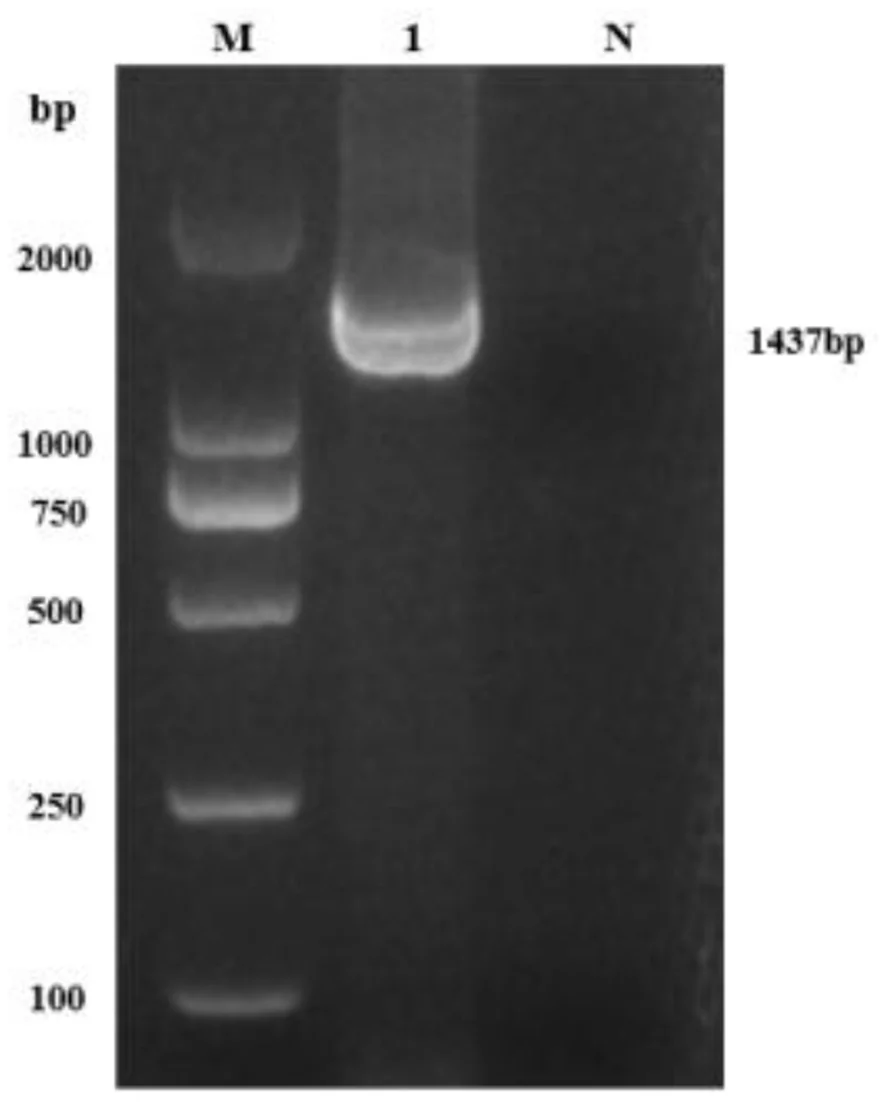
PCR amplification of the 16S rRNA gene of *S. flexneri* from the cynomolgus monkey. (M: DL-2K Marker; 1: sample; N: Negative control).

The amplified PCR product from the isolated strain underwent analysis via 1.5% agarose gel electrophoresis, that revealed a specific target band approximately at 1,500 bp was obtained which consistent with the expected size (Figure 3).

**Figure 4:**
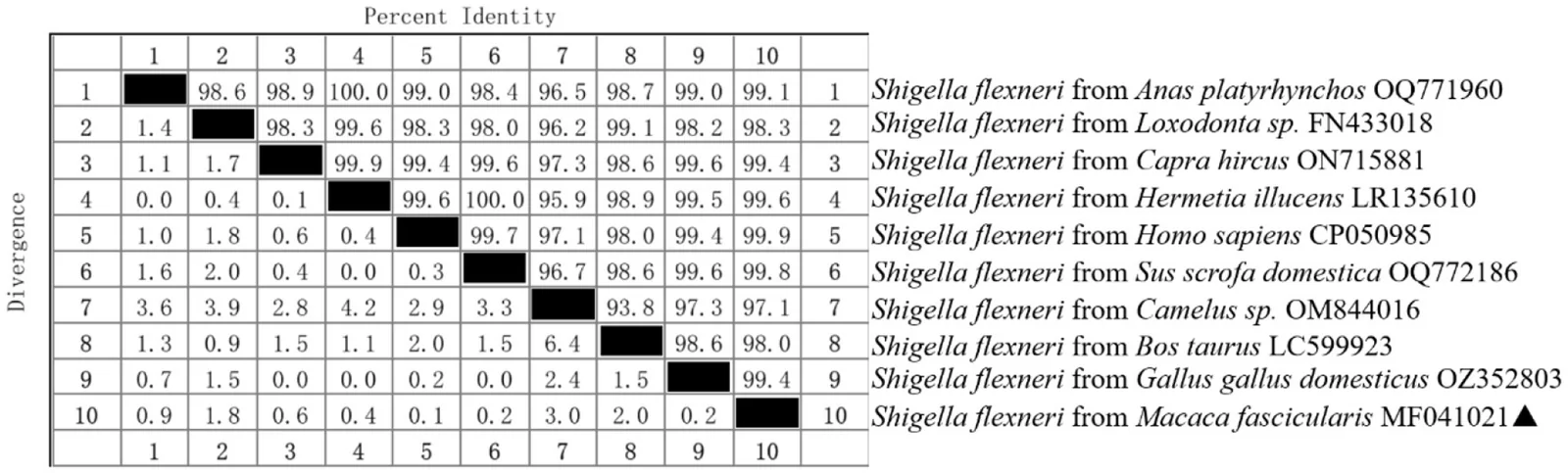
Homology comparison of isolated strains with *S. flexneri* from different sources. “▴” is the isolated bacteria strain from the cynomolgus monkey in this study.

Sequencing identified that the length of the obtained 16S rRNA gene sequence measured at 1,437 bp. NCBI-BLAST sequence alignment confirmed high consistency with *S. flexneri* gene sequences showing up to a remarkable sequence identity rate of 99.9%, thereby confirming that this isolate is indeed *S. flexneri*. Furthermore, comparative sequencing aligned against various *S. flexneri* strains sourced from different hosts available in NCBI GenBank through Clustal W method utilizing MegAlign software illustrated that similarity between the isolated strain MF041021 (PZ779204) and published GenBank sequences exceeded or equaled to over 97.1% similarity (Figure 4), with the highest identity reaching up to 99.9% when compared against human-derived strain (CP050985).

**Figure 5:**
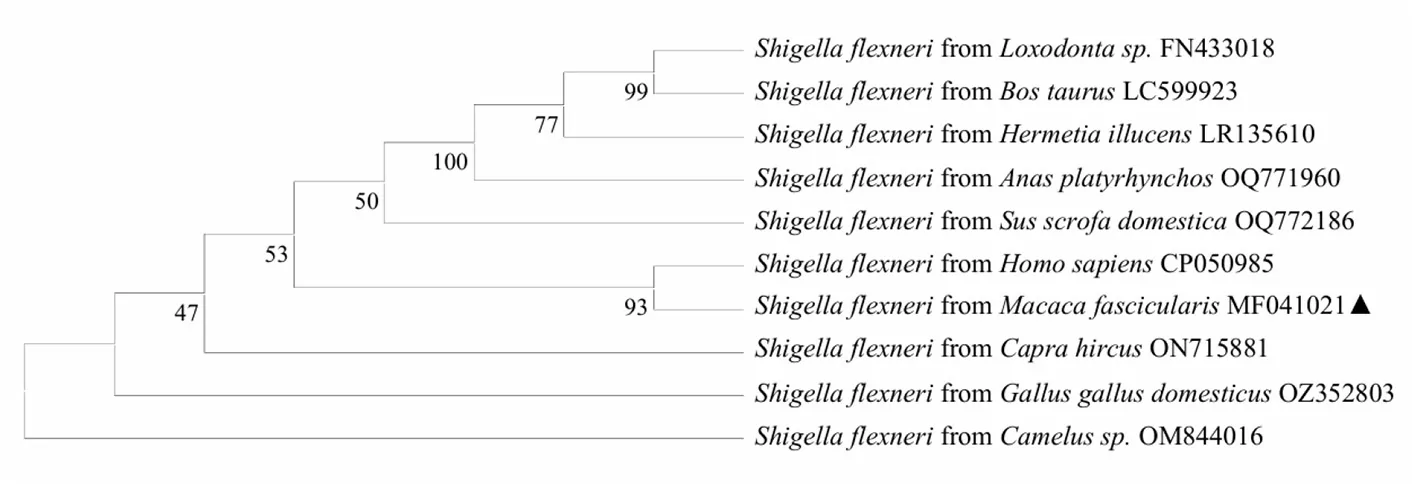
Phylogenetic tree of 16S rRNA sequences among different reported strains of *S. flexneri*, including cynomolgus monkey (*Macaca fascicularis*) (MF041021). “▴” is the isolated bacteria strain from the cynomolgus monkey in this study.

Phylogenetic analysis constructed based on 16S rRNA gene sequences using MEGA11.0 software depicted 10 distinct strains belonging to *S. flexneri* across diverse host origins forming multi-lineage differentiated topologies (Figure 5). Notably, isolate MF041021 (PZ779204) clustered in the same subclade as the human-derived strain CP050985, indicating the closest phylogenetic relationship among all strains analyzed. By contrast, the camel-derived isolate OM844016 formed an independent phylogenetic branch and exhibited the greatest genetic distance from MF041021 (PZ779204).

### 3.5 Detection results of virulence and resistance genes of the isolated strain

**Figure 6:**
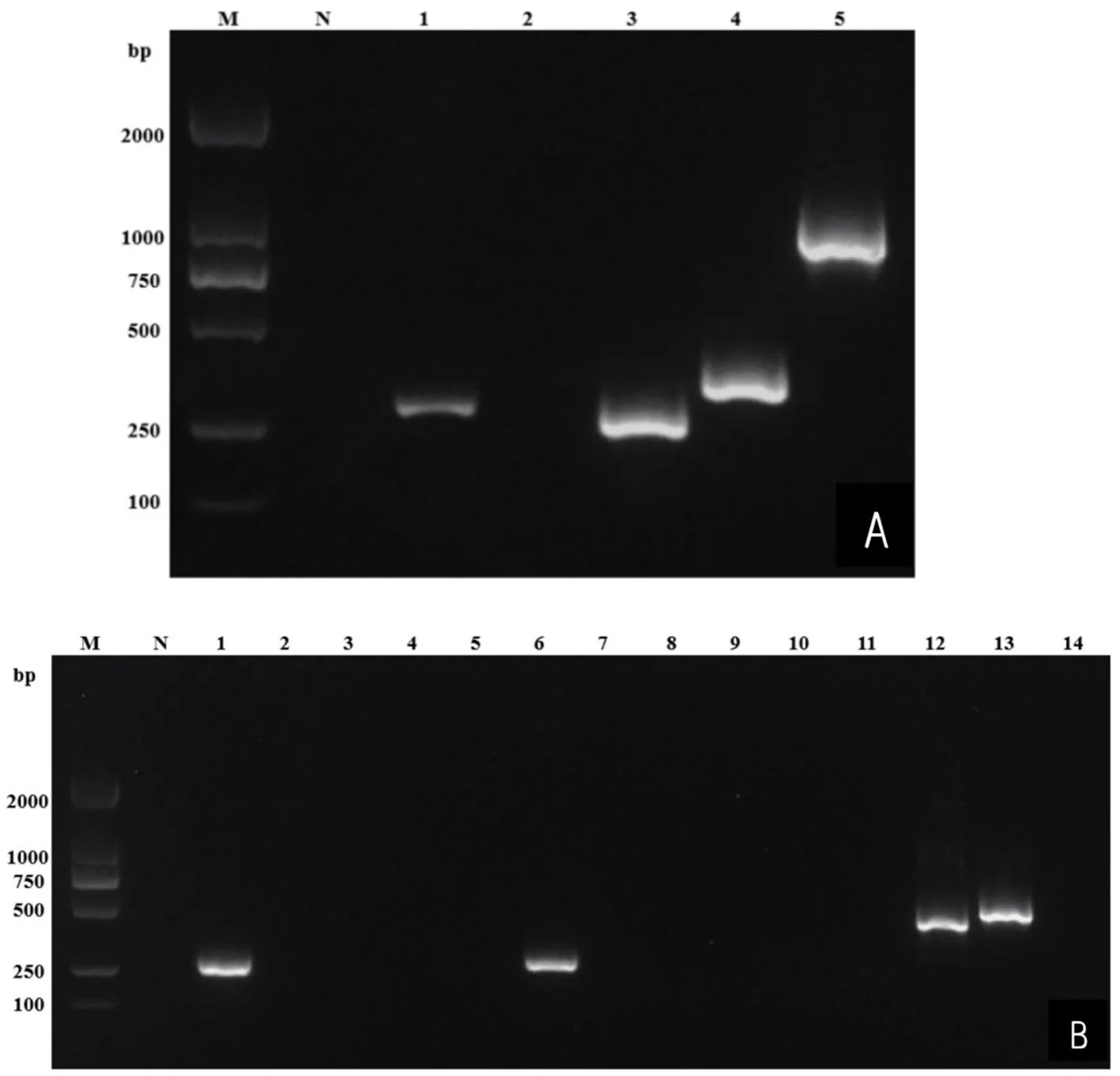
Results of PCR amplification testing for virulence and resistance genes of *S.flexneri*. **(A)** Results of PCR amplification for virulence genes. **(B)** Results of PCR amplification testing for resistance genes. (**A**: M:DL-2K DNA Marker; N: Negative control; 1:*ial*; 2: *sen*; 3:*shet1A*; 4: *ipaH*; 5: *ipaC*). (**B**: M:DL-2K DNA Marker; N:Negative control; 1:*aadA1*; 2*strA-strB*; 3:*rmtB*; 4: *aac(3*′*)-II*; 5: *aph(3*′*)-IIa*; 6: *ant(3*′*)-Ia*; 7:*sul3*; 8:*sul2*; 9:*sul1*; 10:*bla*_*NDM*_; 11:*bla*_*TEM*_; 12:*ampC*; 13:*tetB*; 14:*tetA*).

PCR amplification was conducted to identify common virulence genes and resistance genes of the isolated *S. flexneri*. The results indicated that the isolated strain harbored four virulence genes (*ipaC, ipaH, shet1A, ial*) (Figure 6A) and four resistance genes (*tetB, ampC, ant(3*′*)-Ia, aadA1*) (Figure 6B).

### 3.6 Antimicrobial susceptibility test results of the isolated strain

Antimicrobial susceptibility testing was performed on isolate MF041021 against five clinically relevant antimicrobials, interpreted criteria according to CLSI M100 (2025) for fecal *Shigella* isolates. The isolate was resistant to ampicillin but susceptible to ciprofloxacin, levofloxacin, ofloxacin, and cotrimoxazole. For the exploratory agents, the isolate remained susceptible to imipenem and meropenem, and exhibited a gentamicin inhibition-zone diameter of 17.50 mm (Table 3).

**Table 3:** Results of drug sensitivity testing of *S. flexneri* strains isolated from cynomolgus monkey.

Antibiotics	Medication dose: μg/tablet	IZD/mm (single test)	Judgment standard of inhibition zone diameter (mm)			Sensitivity
			Resistant	Medium sensitivity	Highly sensitive	
Ampicillin	10	7.00	≤ 13	14–16	≥17	R
Ciprofloxacin	5	30.00	≤ 21	22–25	≥26	S
Imipenem	10	34.00	≤ 19	20–22	≥23	S
Meropenem	10	32.00	≤ 19	20–22	≥23	S
Levofloxacin	5	26.00	≤ 16	17–20	≥21	S
Cotrimoxazole	23.75	26.00	≤ 10	11–15	≥16	S
Ofloxacin	5	30.00	≤ 12	13–15	≥16	S
Gentamicin	10 ± 2.5	17.50	NA			NI

S, Highly sensitive; R, Resistant; NA, Not applicable; NI, Not interpreted.

### 3.7 Results of animal pathogenicity tests

All mice subjected to intraperitoneal challenge displayed varying degrees of ruffled fur, decreased feed intake, hunched posture, reduced activity levels, and lethargy. Within 24 h post-challenge, all mice in the high-dose group (4.67 × 10^8^ CFU/mL) succumbed, resulting in a mortality rate of 100%. In contrast, two mice from the low-dose group (4.67 × 10^6^ CFU/mL) died within 72 h following infection, resulting in a mortality rate of 40%, the surviving mice in this group exhibited mild anorexia and lethargy with body tremor after infection, but gradually returned to normal behavior; no significant abnormalities were observed in the control group.

**Figure 7:**
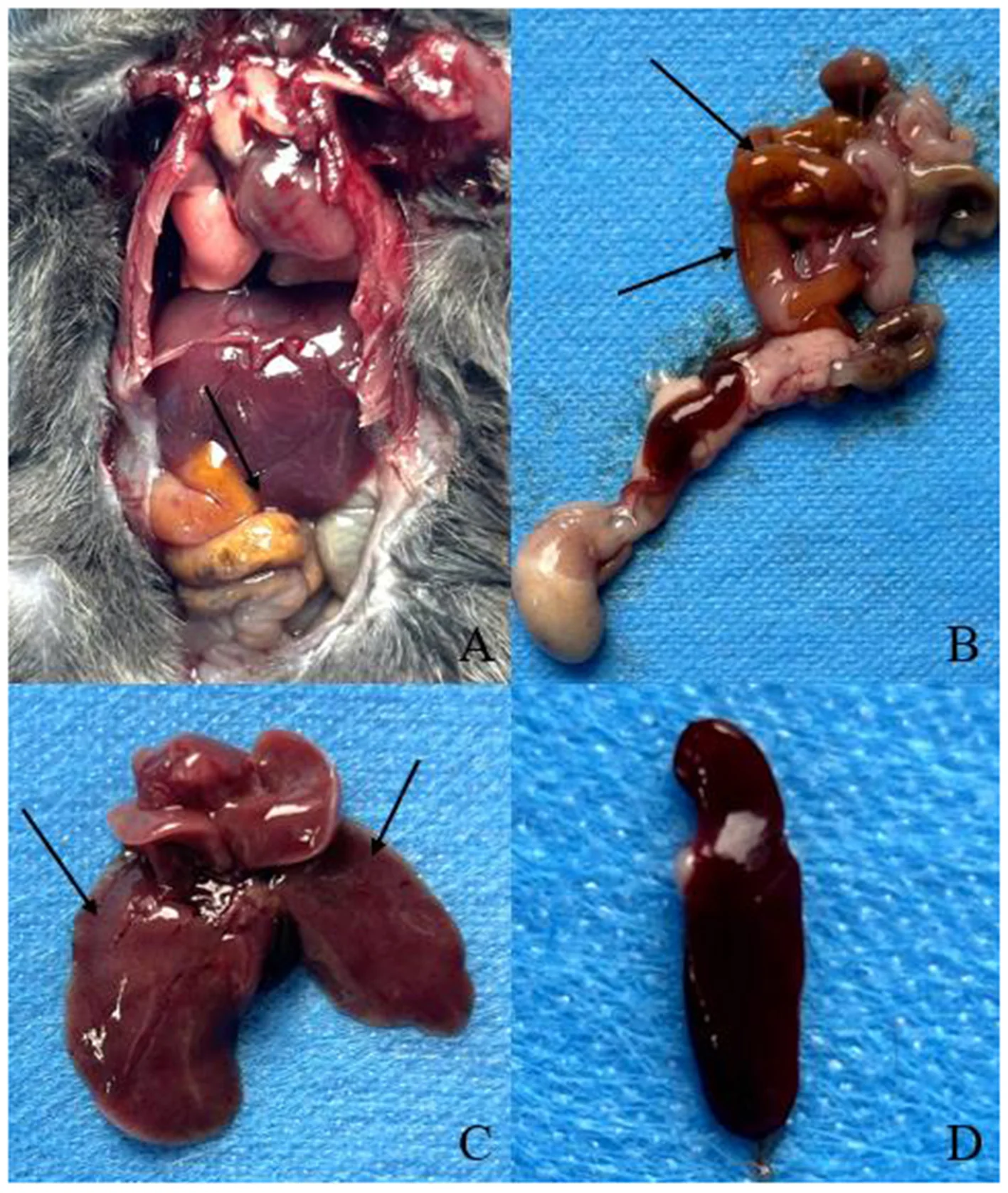
Mouse necropsy findings. **(A)** abdominal cavity. **(B)** gastrointestinal tract. **(C)** liver. **(D)** spleen.

Necropsies performed on deceased mice revealed turbid ascites within the abdominal cavity alongside thinning intestinal wall with diminished tone; substantial yellow fluid accumulation was noted within the intestinal lumen. Additionally, diffuse congestion and hemorrhage were present throughout the intestines; the mucosa of some intestinal segments transitioned from normal light pink to dark brown coloration; the liver appeared enlarged with blunted margins displaying mottled yellowish-brown hues accompanied by pinpoint white necrotic foci on its surface; similarly enlarged spleens presented darker pigmentation (Figure 7). Bacteria were successfully isolated from both liver tissue and cardiac blood samples of deceased mice with molecular identification confirming consistency with the target strain.

## 4 Discussion

*Shigella* is a significant zoonotic pathogen, with primates serving as its natural hosts (Jiang et al., 2023). *Shigella* infections are prevalent in the intestinal tracts of macaques and represent one of the primary pathogenic bacteria (Mannion et al., 2018). The strain isolated from the feces of the diseased cynomolgus monkey was identified as *S. flexneri*. Although none of the five monkeys housed in adjacent cages developed diarrhea and all rectal swab cultures tested negative for *S. flexneri* over the 3-day observation period, these findings should be interpreted with caution. The small sample size, short follow-up duration, and single-time-point sampling are insufficient to rule out asymptomatic carriage, intermittent bacterial shedding, or low-level transmission within the colony. These possibilities warrant further longitudinal investigation, particularly given that *S. flexneri* pathogenesis involves complex interactions between multiple virulence determinants and host factors (Pakbin et al., 2023). Previous studies have shown that a hallmark of *S. flexneri* pathogenicity is its ability to invade intestinal epithelial cells and disseminate via cell to cell spread, processes involving the type III secretion system (T3SS): initially, the bacteria adhere to intestinal epithelial cells via fimbriae, subsequently inject effector proteins through T3SS to invade these cells, and utilize IcsA protein to recruit host actin for intracellular motility and intercellular dissemination (Qin et al., 2020; Mattock and Blocker, 2017), ultimately disrupting the cytoskeleton and intestinal epithelial barrier, leading to inflammation and ulceration (Schroeder and Hilbi, 2008). Membrane protrusions and double-membrane vesicles formed during infection serve as critical morphological bases for this spread, which has been corroborated by electron microscopy observation in rhesus monkey models (Takeuchi et al., 1968) and dynamic capture using *in vivo* fluorescence microscopy (Dragoi and Agaisse, 2014, 2015). Furthermore, *Shigella* toxin can induce additional cytotoxicity, enterotoxicity, and neurotoxicity, resulting in severe complications such as intestinal perforation, hyponatremia, and hemolytic uremic syndrome (Lee et al., 2016). Virulence-related genes of *Shigella* are primarily categorized into two groups: one group encompasses genes associated with adhesion and invasion of intestinal epithelial cells-specifically *ipaH* gene encoding invasion plasmid antigen H; the ipa operon (including *ipaC*, etc.,) on the virulence plasmid encodes Ipa proteins essential for bacterial invasion (Mavris et al., 2002); additionally, the *ial* gene located on the large invasion plasmid also participates in this process (Malau et al., 2018). The second group includes genes that encode *Shigella* enterotoxin: *shet1A* gene on chromosome encodes *Shigella* enterotoxin SHET1 capable of inducing intestinal fluid accumulation in rabbit ileal loops (Fasano et al., 1997); *sen* gene encodes enterotoxin SHET2 (Roy et al., 2006). In this study, we selected five aforementioned virulence genes for PCR detection of the isolated strain. Results indicated that four virulence genes (*ipaC, ipaH, ial, shet1A*) were present while *sen* gene was not detected. This suggests that although there exists a complete profile related to invasion-associated genes along with SHET1 enterotoxin gene, while the absence of the SHET2 enterotoxin gene (*sen*) indicates that its pathogenicity may predominantly rely on these factors consistent with observed strong pathogenicity alongside diarrhea symptoms noted during animal experiments. In this study, a mouse intraperitoneal inoculation model was employed to assess the pathogenicity of *S. flexneri* MF041021. Intraperitoneal inoculation represents a classic *in vivo* approach for assessing bacterial virulence and is suitable for evaluating the systemic invasiveness and virulence of bacterial strains. However, because *S. flexneri* primarily causes intestinal infection via the oral route, this model cannot fully simulate the initial host–pathogen interactions at the intestinal mucosa or the natural progression of disease. Future studies may employ oral infection models or non-human primate challenge models to further elucidate the mechanisms underlying intestinal colonization, mucosal invasion, and *in vivo* pathogenesis.

In recent years, growing cases of *Shigella* infection have been documented in a wide range of animal hosts, including rhesus monkey (Kim et al., 2023), yak, beef (Liang et al., 2022) and housefly (Mudau et al., 2023), suggesting a continuous expansion of the host spectrum of this pathogen. While extensive studies have focused on *S. flexneri*, dysentery induced by *S. flexneri* in cynomolgus monkeys has been rarely reported. Other studies in the literature have clearly indicated that the genetic divergence of 16S rRNA genes between *S. flexneri* and *Escherichia coli* (*E. coli*) K-12 is approximately 1.5% (Lan and Reeves, 2002). Accumulated evolutionary evidence has verified that *Shigella* originates from non-invasive *E. coli* strains (Pupo et al., 2000; Yang et al., 2007). This evolutionary adaptation is primarily driven by the acquisition of critical virulence factors via horizontal gene transfer, accompanied by the deletion of functional genes such as *cadA* and *ompT* through reductive genome evolution, which enhances the pathogenicity of *Shigella* strains (Ochman et al., 2000). The phylogenetic tree constructed based on 16S rRNA gene sequences demonstrated that the isolated strain MF041021 shared 97.1–99.9% nucleotide identity with nine reference *S. flexneri* strains derived from diverse animal and human hosts deposited in the GenBank database. Notably, strain MF041021 exhibited the highest sequence similarity (99.9%) to a human-origin *S. flexneri* isolate (CP050985). Phylogenetic analysis also revealed obvious multilineage differentiation among the 10 *S. flexneri* strains from different hosts. Specifically, strain MF041021 clustered within the same genetic evolutionary clade with six strains of *S. flexneri* isolated from human (CP050985), pig (OQ772186), duck (OQ771960), black soldier fly (LR135610), cattle (LC599923) and elephant (FN433018), and showed the closest phylogenetic relationship with the human-derived strain. By contrast, three strains isolated from goat (ON715881), chicken (OZ352803) and camel (OM844016) formed three relatively independent evolutionary branches. Collectively, these phylogenetic results confirmed that the strain MF041021 had the closest genetic relationship to the human-derived strain (CP050985) and the farthest relatedness to the camel-derived strain (OM844016). These findings are consistent with the recognized susceptibility of nonhuman primates to *S. flexneri* infection. Nevertheless, 16S rRNA gene-based phylogenetic analysis revealed that the monkey-derived isolate MF041021 was closely genetic relatedness with human-derived pathogenic strains. However, since no human, environmental, or additional animal isolates were included in the present study, this phylogenetic relationship should not be construed as direct evidence of cross-host transmission, endemic circulation, or epidemiological linkage. Future comparative studies incorporating isolates from humans, non-human primates, other animal species, and relevant environmental sources, together with whole-genome and epidemiological investigations, will be required to delineate the potential transmission dynamics and reservoir hosts of *S. flexneri*. In this study, 16S rRNA gene sequencing was employed for preliminary molecular identification and phylogenetic placement of the isolate. However, because *Shigella* spp. and *Escherichia coli* are closely evolutionary relationship and share highly similar 16S rRNA gene sequences, this marker exhibits limited discriminatory power for distinguishing these taxa and resolving strain-level phylogenetic relationships. Consequently, 16S rRNA gene analysis alone is insufficient for definitive species identification or high-resolution phylogenetic inference. Nevertheless, the identification of isolate MF041021 as *S. flexneri* was corroborated by colony morphology, biochemical characterization, and serotyping. Future studies should employ whole-genome sequencing, together with core-genome single-nucleotide polymorphism analysis, where applicable, multilocus sequence typing (MLST), to achieve finer phylogenetic relationships of strain MF041021 and comprehensively characterize its virulence and antimicrobial resistance determinants. When integrated with broader comparative sampling and epidemiological data, these approaches will also facilitate assessment of potential epidemiological relationships between isolates from diverse sources. Antibiotic therapy significantly impacts bacillary dysentery caused by *Shigella*, effectively reducing both mortality and infection rates. However, the widespread use of broad-spectrum antibiotics has led to a steady increase in antimicrobial resistance among *S. flexneri* strains. Under the selective pressure exerted by these antibiotics, *Shigella* has acquired invasive virulence factors and immune evasion capabilities (such as inducing macrophage apoptosis), along with resistance loci like SRL through horizontal gene transfer, resulting in multidrug resistance (Ochman et al., 2000). Consequently, the high pathogenicity observed in contemporary *Shigella* is fundamentally a product of the co-evolution between its invasive virulence mechanisms, immune evasion strategies, and intrinsic antimicrobial resistance. Currently, there is an ongoing rise in antimicrobial resistance among strains while clinical efficacy of available antibiotics continues to diminish. Historically sensitive to common antibiotics such as nalidixic acid, trimethoprim-sulfamethoxazole, chloramphenicol and ampicillin, *Shigella* has now developed resistance against cephalosporins, fluoroquinolones and macrolides such as erythromycin and azithromycin (Satija and Anjankar, 2024; Shooraj et al., 2024). In this study, isolate MF041021 exhibited resistance to ampicillin but was susceptible to ciprofloxacin, levofloxacin, ofloxacin, and cotrimoxazole. The isolate also showed susceptibility to both imipenem and meropenem. However, clinical evidence supporting carbapenems use for shigellosis remains limited, and these agents are generally reserved for cases where first-line therapeutic options are ineffective due to resistance. Nevertheless, their clinical importance in managing multidrug-resistant Enterobacterales infections justified their inclusion in this exploratory assessment. Aminoglycosides are not routinely recommended by CLSI for clinical reporting of fecal *Shigella* isolates. Accordingly, gentamicin was included solely to explore potential genotype–phenotype associations, and an inhibition-zone diameter of 17.50 mm was recorded without assigning a clinical susceptibility category. PCR analysis identified the β-lactam resistance gene *ampC*, the aminoglycoside resistance determinants *ant(3*′*)-Ia* and *aadA1*, and the tetracycline resistance gene *tet(B)*. The presence of these genes indicates a potential genetic basis for antimicrobial resistance; however, their phenotypic expression and contribution to the observed susceptibility profile require further functional investigation.

From a mechanistic perspective on antibiotic resistance, the *ampC* gene encodes AmpC β-lactamase which hydrolyzes the β-lactam ring present in penicillin drugs such as ampicillin leading to their deactivation (Jacoby, 2009); this finding aligns with the strain's observed phenotype of ampicillin resistance. The *ant(3*′*)-Ia* and *aadA1* genes encode aminoglycoside-modifying enzyme (ANT) and adenylyltransferase (AAD), respectively (Ramirez and Tolmasky, 2010), which reduce antibacterial activity against aminoglycoside drugs via adenylylation modification. Notably, although aminoglycoside resistance genes were detected, the inhibition-zone diameter observed for gentamicin suggested that it may retain *in vitro* activity against the isolate. This apparent inconsistency may be attributable to differences in the substrate specificities of individual aminoglycoside resistance determinants. Specifically, *aadA1* and *ant(3*′*)*-*Ia* primarily confer resistance to streptomycin and spectinomycin, with minimal impact on gentamicin susceptibility (Stern et al., 2018; Saleh et al., 2023). Consistent with this, previous studies have also reported that the presence of *aadA1* does not necessarily correlate with a gentamicin-resistant phenotype (Xu et al., 2013).

The *tetB* gene encodes a tetracycline efflux protein mediating tetracycline resistance through an active efflux mechanism (Grossman, 2016). However, tetracycline susceptibility testing was not performedin this study; therefore, it remains unclear whether the gene is expressed and confers the corresponding resistance phenotype, which represents a limitation of this study. Compared with the broader antimicrobial resistance trends reported in previous studies, the relatively narrow resistance profile of this isolate may reflect the strict and standardized husbandry practices implemented at our facility. These include rigorous environmental biosecurity control, a systematic antibiotic usage strategy, and a relatively closed colony management system. However, this interpretation remains speculative, as these factors were not directly assessed in the present study, and further epidemiological investigations are required to substantiate this hypothesis.

This study presents a comprehensive biological characterization of *S. flexneri* isolated from a diseased cynomolgus monkey. Although the findings are inherently limited by the single-isolate design, this in-depth case analysis offers a valuable reference for elucidating host-pathogen interactions, assessing local epidemiological risks, and optimizing biosecurity management in laboratory animal facilities. Further investigations with larger sample sizes are warranted to validate and extend these findings.

## 5 Conclusion

In conclusion, this study successfully isolated *S. flexneri* from the feces of a cynomolgus monkey exhibiting dysentery and performed comprehensive characterization that included morphological observation, biochemical identification, 16S rRNA gene sequencing with phylogenetic analysis, detection of virulence and antimicrobial resistance genes, animal pathogenicity assays, as well as antimicrobial susceptibility testing. These findings provide valuable reference data for accurate identification and clinical management of *S. flexneri* infections in captive laboratory macaques while holding practical significance for enhancing health quality among non-human primates utilized in biomedical research. It is important to note that the conclusions of this study are based primarily on analysis of a single isolate; larger-scale studies are warranted to validate and extend these findings.

## Data Availability

The datasets presented in this study can be found in online repositories. The names of the repository/repositories and accession number can be found below: NCBI GenBank, accession number PZ779204. Further inquiries can be directed to the corresponding author.
